# Increased Risk for Malignancies in 131 Affected *CTLA4* Mutation Carriers

**DOI:** 10.3389/fimmu.2018.02012

**Published:** 2018-09-10

**Authors:** David Egg, Charlotte Schwab, Annemarie Gabrysch, Peter D. Arkwright, Edmund Cheesman, Lisa Giulino-Roth, Olaf Neth, Scott Snapper, Satoshi Okada, Michel Moutschen, Philippe Delvenne, Ann-Christin Pecher, Daniel Wolff, Yae-Jean Kim, Suranjith Seneviratne, Kyoung-Mee Kim, Ji-Man Kang, Samar Ojaimi, Catriona McLean, Klaus Warnatz, Maximilian Seidl, Bodo Grimbacher

**Affiliations:** ^1^Faculty of Medicine, Center for Chronic Immunodeficiency, Medical Center of the University Hospital, University of Freiburg, Freiburg, Germany; ^2^Royal Manchester Children's Hospital, University of Manchester, Manchester, United Kingdom; ^3^Division of Pediatric Hematology/Oncology, Department of Pediatrics, Weill Cornell Medicine, New York, NY, United States; ^4^Seccion de Infectologia e Inmunopatologia, Unidad de Pediatria, Hospital Virgen del Rocio/Instituto de Biomedicina de Sevilla, Sevilla, Spain; ^5^Division of Pediatric Gastroenterology, Hepatology, and Nutrition, Department of Medicine, Children's Hospital Boston, Boston, MA, United States; ^6^Department of Pediatrics, Hiroshima University Graduate School of Biomedical & Health Sciences, Hiroshima, Japan; ^7^Department of Infectious Diseases and General Internal Medicine, University Hospital of Liege, Liege, Belgium; ^8^Department of Internal Medicine II, University Hospital Tübingen, Tübingen, Germany; ^9^Department of Internal Medicine III, University Hospital Regensburg, Regensburg, Germany; ^10^Division of Infectious Diseases and Immunodeficiency, Samsung Medical Center, Sungkyunkwan University School of Medicine, Seoul, South Korea; ^11^Institute of Immunity and Transplantation, Royal Free Hospital, University College London, London, United Kingdom; ^12^Department of Pathology & Translational Genomics, Samsung Medical Center, Sungkyunkwan University School of Medicine, Seoul, South Korea; ^13^National Cancer Center, Goyang, South Korea; ^14^Department of Paediatrics, Monash University, Clayton, VIC, Australia; ^15^Alfred Health, Prahran, VIC, Australia

**Keywords:** CTLA4, combined immunodeficiency, primary immunodeficiency, malignancy, cancer predisposition, EBV, CMV

## Abstract

**Background:** Cytotoxic T-lymphocyte-associated antigen 4 (CTLA-4) is a negative immune regulator on the surface of T cells. In humans, heterozygous germline mutations in *CTLA4* can cause an immune dysregulation syndrome. The phenotype comprises a broad spectrum of autoinflammatory, autoimmune, and immunodeficient features. An increased frequency of malignancies in primary immunodeficiencies is known, but their incidence in CTLA-4 insufficiency is unknown.

**Methods:** Clinical manifestations and details of the clinical history were assessed in a worldwide cohort of 184 *CTLA4* mutation carriers. Whenever a malignancy was reported, a malignancy-specific questionnaire was filled.

**Results:** Among the 184 *CTLA4* mutation carriers, 131 were considered affected, indicating a penetrance of 71.2%. We documented 17 malignancies, which amounts to a cancer prevalence of 12.9% in affected *CTLA4* mutation carriers. There were ten lymphomas, five gastric cancers, one multiple myeloma, and one metastatic melanoma. Seven lymphomas and three gastric cancers were EBV-associated.

**Conclusion:** Our findings demonstrate an elevated cancer risk for patients with CTLA-4 insufficiency. As more than half of the cancers were EBV-associated, the failure to control oncogenic viruses seems to be part of the CTLA-4-insufficient phenotype. Hence, lymphoproliferation and EBV viral load in blood should be carefully monitored, especially when immunosuppressing affected *CTLA4* mutation carriers.

## Introduction

Antibody-mediated autoimmunity, recurrent infections, lymphoproliferation, and lymphoid infiltrations into the lung, the bowel, or the central nervous system, characterize the clinical spectrum of CTLA-4-insufficient patients (1, 2). Furthermore, recent publications reported on malignancies in those patients (3–6).

Cytotoxic T-lymphocyte-associated antigen 4 (CTLA-4) is an essential negative regulator of T cell-mediated immune responses (7). CTLA-4 competes with CD28 with higher affinity for the binding to CD80 and CD86 (also known as B7-1 and B7-2), two costimulatory molecules on the surface of dendritic cells, activated B cells, and monocytes. CD28 is constitutively expressed on T cells whereas CTLA-4 is mostly stored in vesicles and becomes upregulated in conventional T cells upon activation. Whereas CD28 delivers a positive signal into T cells leading to T cell activation and effector cell differentiation, the ligation of CTLA-4 to CD80 and CD86 delivers a negative signal to T cells, limiting IL-2 production, proliferation, and survival of T cells (8). Thus, heterozygous *CTLA4* germline mutations impair the suppressive function of T cells and cause an immune dysregulation syndrome characterized by an activated immune system with autoimmune features and organ pathology caused by infiltrating effector T cells (2).

Given that patients with heterozygous *CTLA4* mutations have an activated immune system consisting of an expansion of effector T cells and given that anti-CTLA-4 treatment is successfully used in the treatment of certain cancers, one would expect that patients with CTLA-4 insufficiency have a lower risk to develop cancer (2, 6, 9). Hence, our study aimed to assess whether a heterozygous germline mutation in *CTLA4* is associated with a decreased risk to develop cancer.

## Materials and methods

We have collected a world-wide cohort of 184 *CTLA4* mutation carriers. Of these, 131 were considered affected (mutation carriers were classified as affected if they showed clinically apparent symptoms related to CTLA-4 insufficiency requiring medical care or treatment), indicating a reduced penetrance of the disease of 71.2%. A malignancy-specific questionnaire was provided to collaborating physicians, who had reported any malignancy in their CTLA-4-insufficient patients, to collect data on cancer entity, cancer induction, and treatment outcome. This study was carried out in accordance with the recommendations for studies with human subjects of the scientific committee at the University Medical Center of Freiburg. All physicians confirmed that their patients had signed an informed consent under local ethics-approved protocols and in accordance with the Declaration of Helsinki. The protocol was approved by the scientific committee at the University Medical Center of Freiburg (Approval No. 251/13_KW and 295/13_140782).

The following patients are part of a single-center retrospective analysis of malignancy cases associated with common variable immunodeficiency (CVID) as well: A.III.1 = ID 21, MM.II.1 = ID 7, and JJ.II.1 = ID 13.

All statistics and figures were computed and created by using R (version 3.4.1). Person-time of risk described the time period from date of birth to last follow-up or until an event (cancer or death) occurred and was used to compute life-time incidence rate. Individuals were censored, if follow-up date was missing (six individuals). Incidence rates of malignancies in *CTLA4* wildtype population are in cases per 100,000 persons per year and are age-adjusted to the 2000 US Std Population (19 age groups—Census P25-1130 for US rates), respectively to the SEGI-world standardization for German rates and were arithmetically averaged (10, 11).

## Results

We identified 17 patients with a malignancy (Table 1). This amounts to a cancer prevalence of 12.9% in patients with CTLA-4 insufficiency in our cohort. Among the 17 cases there were two entities predominant: ten patients with lymphomas and five patients with gastric cancers. Interestingly, seven of ten lymphomas and three of five gastric cancers were EBV-associated. Additionally, one case of multiple myeloma, and one case of metastatic melanoma were reported.

**Table 1:** CTLA-4-insufficient-patients with malignancies, viral association, and treatment details.

									**Treatment**				
**Cohort ID**	**Sex**	***CTLA4* Mutation**	**Type of malignancy**	**Age of diagnosis of malignancy**	**CVID diagnosis**	**EBV pos**.	**CMV pos**.	**Other pos**.	**Chemotherapy**	**Surgery**	**HSCT**	**Others**	**Remission**
**GROUP ONE—LYMPHOMAS**													
A.III.1	Female	c.105C>A; p.C35^*^	Hodgkin lymphoma	30	Yes	Yes	No	No	AVD-Brentuximab	No	No	No	Pending
B.II.3	Male	c.109+1G>T	Hodgkin lymphoma	51	No	Yes	No	No	A(B)VD	No	Yes	No	Yes
H.II.1	Male	c.407C>T; p.P136L	Hodgkin lymphoma	21	No	Yes	No	No	RTX, Vincristine	No	No	No	No
L.II.2	Male	c.437G>T; p.G146V	Hodgkin lymphoma	17	No	Yes	No	No	ABVD	No	Yes	No	Yes
JJ.II.1	Male	c.223C>T; p.R75W	Hodgkin lymphoma	28	Yes	Yes	No	No	ABVD	No	No	No	Yes
MM.II.1	Male	c.530_543del; p.F179Cfs*29	Hodgkin lymphoma	33	Yes	Yes	No	No	ABVD	No	No	No	Yes
K.II.1	Female	c.308G>C; p.C103S	DLBCL	45	No	No	No	No	R-CHOP, R-DHAP, R-BEAM	No	No	No	No
UU.III.3	Female	§	DLBCL	50	No	No	No	No	R-CHOP	No	No	Yes	No
CO.I.1	Male	c.209G>A; p.R70Q	DLBCL	62	Yes	No	No	No	R-CHOP	No	No	No	No
FF.II.1	Male	c.208C>T; p.R70W	Burkitt lymphoma	22	No	No	No	No	R-CVAD, R-ICE, DA-R-EPOCH	No	No	No	No
**GROUP TWO—GASTRIC CANCERS**													
B.II.4	Female	c.109+1G>T	Gastric adenocarcinoma	40	No	Yes	No	No	No	Yes	No	No	Yes
G.III.2	Male	c.179A>G; p.Y60C	Gastric adenocarcinoma	17	No	No	No	No	No	Yes	No	No	Yes
M.II.3	Male	c.76_77insT; p.F28Sfs*40	Gastric adenocarcinoma	34	Yes	No	Yes	Hp	No	Yes	No	No	No
XX.II.1	Male	c.407C>T; p.P136L	Gastric adenocarcinoma	42	Yes	Yes	Yes	Hp	No	No	No	Yes	Yes
CM.II.2	Female	c.406C>T; p.P136S	Gastric adenocarcinoma	25	Yes	No	No	No	No	Yes	No	No	Yes
**GROUP THREE—OTHER MALIGNANCIES**													
CM.I.2	Male	c.406C>T; p.P136S	Multiple myeloma	75	No	No	No	No	Bortzezomib, Melphalan	No	No	No	Pending
CZ.II.2	Female	c.410C>T; p.P137L	Metastatic melanoma	24	No	No	No	No	No	Yes	No	Yes	Pending

*HP, Helicobacter pylori; DLBCL, Diffuse large B cell lymphoma; CVID, Common variable immunodeficiency; pos, positive; chemotherapy abbreviations, see Abbreviations section; remission no, deceased; ^§^Deceased prior being genotype*.

* *translation termination codon*.

The cumulative person-years of documentation in our cohort of 184 *CTLA4* mutation carriers comprise 3,825 patient-years in total. Taken together, the reported 17 cases result in 4.4 new malignancies per 1,000 patients per year.

The median age of onset was 32 years for lymphoma and 34 years for gastric cancer. The Hodgkin lymphomas occurred at a median age of 29 years and the Non-Hodgkin-Lymphomas at a median age of 48 years. The multiple myeloma occurred at the age of 75 years and the melanoma at the age of 24 years. The median age of death was 53 years for DLBCL, 35 years for gastric cancer, and 43 years in total.

Six of the 10 lymphomas were classified as Hodgkin lymphomas, of whom one patient is currently on treatment (A.III.1), four patients reached a complete remission (B.II.3, L.II.2, JJ.II.1, MM.II.1), and one patient died (H.II.1). Two out of four patients who were cured received chemotherapy (A(B)VD protocol) followed by a successful allo-HSCT (B.II.3 and L.II.2).

Four of ten lymphomas were classified as Non-Hodgkin lymphomas (NHL): Three diffuse large B cell lymphomas (DLBCL) in K.II.1, UU.III.3, and CO.I.1, and one Burkitt lymphoma in FF.II.1. All four patients died due to their NHL.

Additionally, one patient (CM.I.2) developed a multiple myeloma and is currently on treatment. All details in Table 1.

The five patients with gastric cancer had a long history of autoimmune enteropathy or atrophic gastritis. Three of the gastric carcinomas were EBV-associated, two were positive for CMV and *Helicobacter pylori* (Table 1). Three of the patients have previously been published (G.III.2, M.III.3 and B.II.4) (2, 4, 5). For treatment, three out of five patients underwent total gastrectomy, one received subtotal gastrectomy, and one patient (XX.II.1) underwent endoscopic resection and stopped his recent abatacept therapy. The first gastroscopy after 6 months in patient XX.II.1 showed quiescent conditions and gastrectomy might finally be performed when aggravation occurs. So far, remission was reached in four patients, however, patient M.II.3 never fully recovered and died 25 months after gastrectomy.

The two further malignancies comprise one patient (CZ.II.2), who had developed a metastatic melanoma and precancerous cervical and vulvar lesions, indicating decreased control of Human papilloma viruses (HPV). Excision of the melanoma was performed, but suspicious PET-CT avidity prompted further interventions of which results are currently pending. The second patient (CM.I.2) had developed an axillary sarcoma at the age of 60 years, had reached clinical remission, but had presented recently an amyloidosis-associated multiple myeloma at the age of 75 years and is currently on treatment.

**Figure 1:**
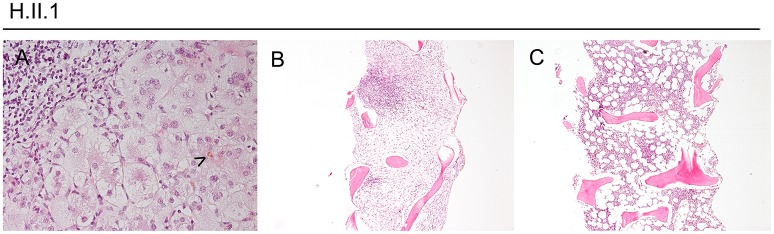
Hyperinflammation, exemplified in a CTLA-4-insufficient-patient. Panel **(A)** shows a liver biopsy taken from patient H.II.1 at the age of 17 years with a cholestatic giant cell hepatitis (bile marked by arrowhead, exemplary). Panels **(B,C)** show bone marrow trephine biopsies with nodular lymphocytic aggregates (of T-cell origin, by IHC). In **(B)**, subtotal replacement of hematopoiesis by fibrosis and stromal edema can be seen.

**Figure 2:**
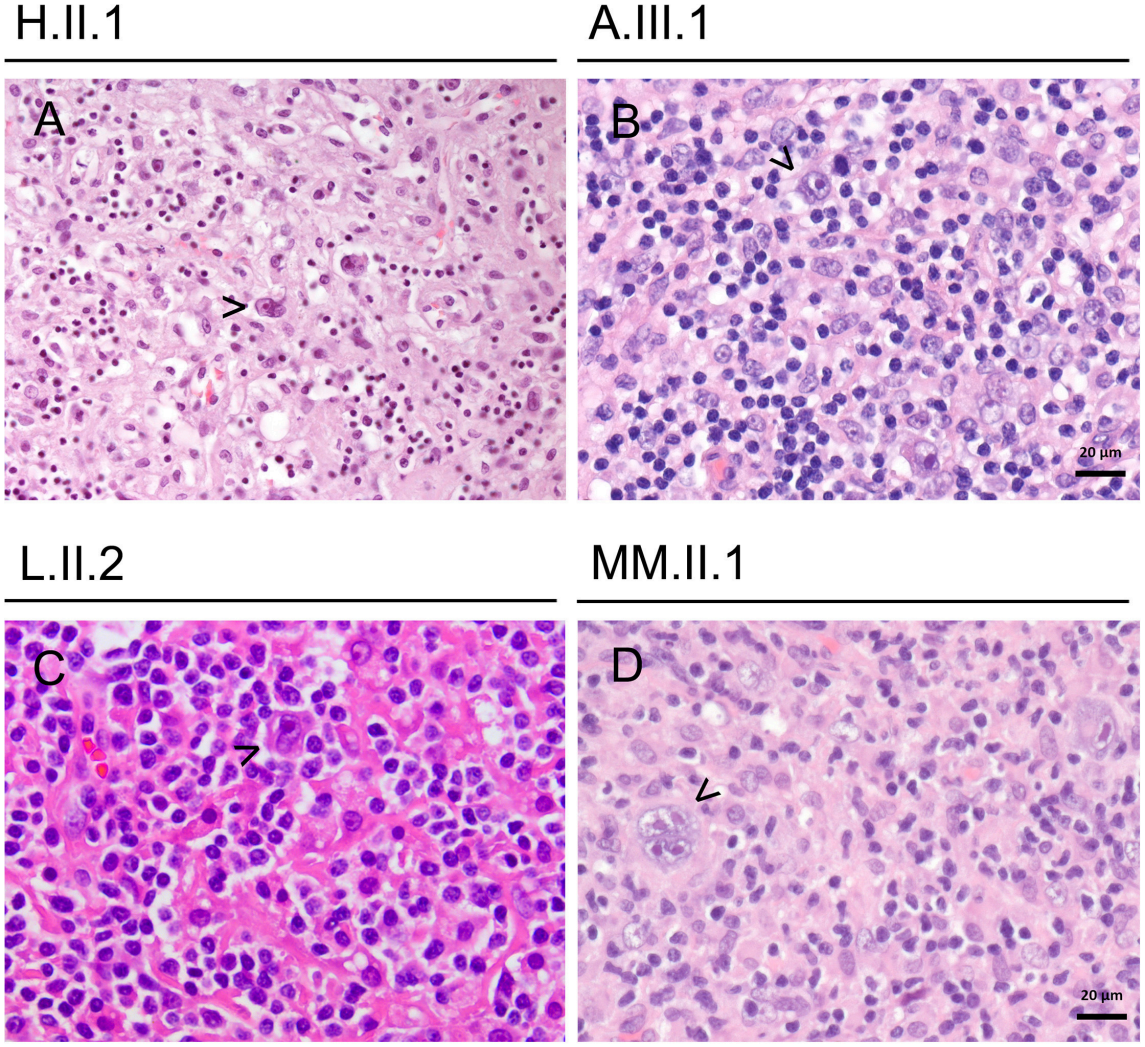
Lymphomas in CTLA-4-insufficient-patients **(A–D)** showing classical Hodgkin lymphomas (mixed cellularity subtype), exemplary Reed-Sternberg cells are highlighted by arrowhead. All cases were EBV-associated.

**Figure 3:**
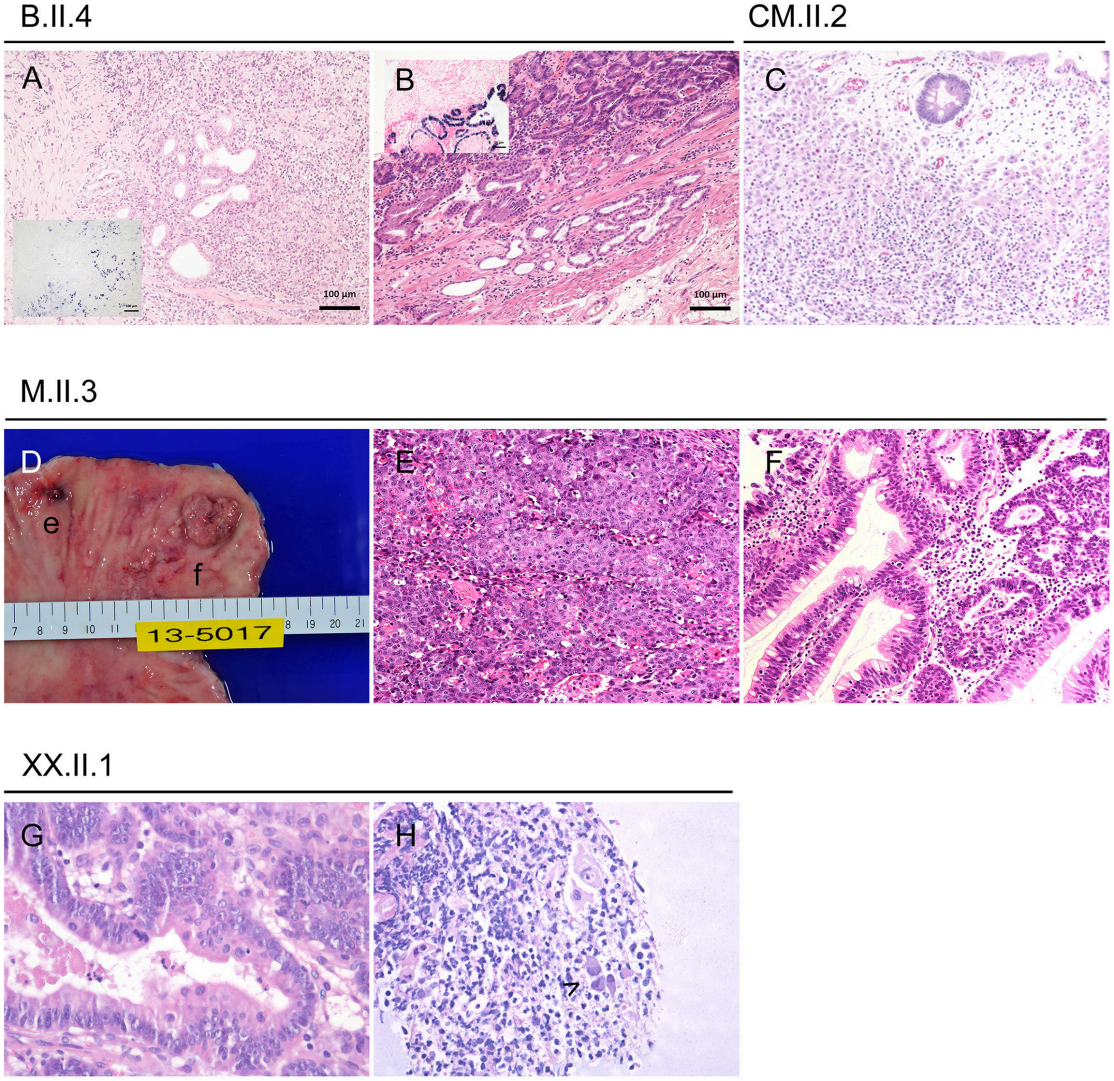
Gastric cancer and precursor lesions in CTLA-4-insufficient-patients. **(A)** Poorly differentiated EBV associated adenocarcinoma at the age of 41 years (inlet displays chromogen *in situ* hybridization of EBV coded mRNA/EBER ISH). **(B)** Same patient at the age of 44 years with a well-differentiated EBV associated adenocarcinoma (inlet with EBER ISH). **(C)** Infiltration of the lamina propria through discohesive carcinoma cells in a 25-years-old patient. (D-F) 34-years-old patient with small poorly differentiated adenocarcinoma (**D**,e for macroscopy; **E** for histology) and larger well-differentiated adenocarcinoma (**D**,f for macroscopy; **F** for histology). **(G,H)** Pyloric biopsies taken at the age of 43 years showing high grade dysplasia **(G)** and viral inclusions (**H**, highlighted by arrowhead).

**Figure 4:**
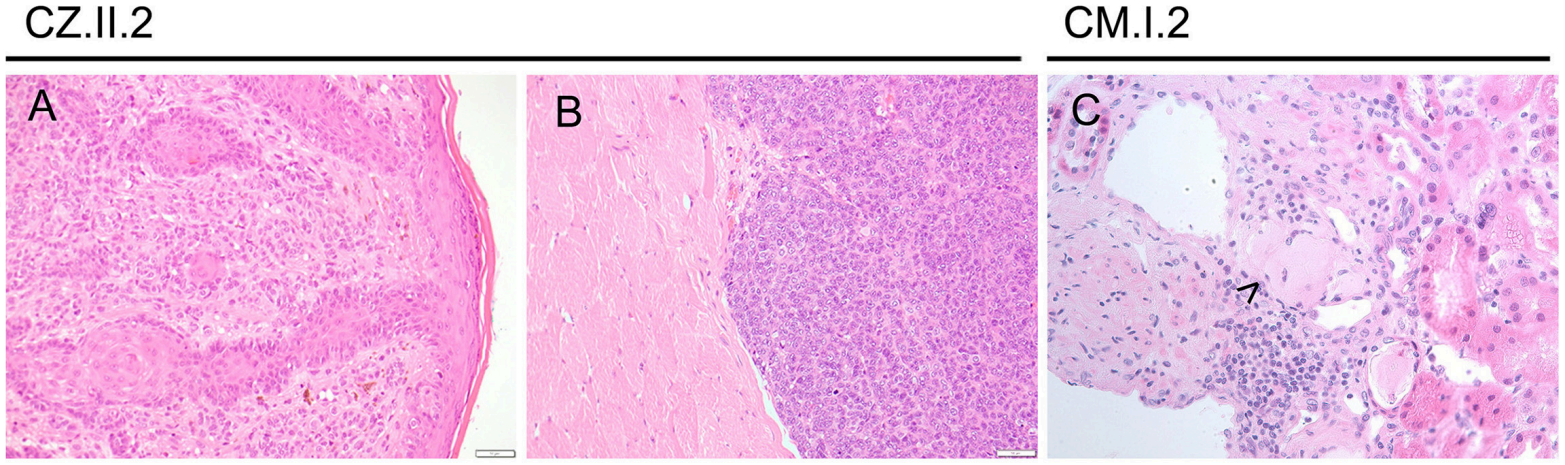
Other malignancies in CTLA-4-insufficient-patients. **(A)** Superficial spreading melanoma of the ear (at the age 24 years) and metastasis (**B**; at the age of 25 years) infiltrating striated muscle fibers. **(C)** Amyloid deposits (highlighted by arrowhead) were immunohistochemically positive for amyloid p in this 75-years-old patient with multiple myeloma.

Histologically, the hyperinflammatory condition of CTLA-4-insuffient patients often manifests at mucosal surfaces of the gastrointestinal tract but may also damage associated organs as the liver (Figure 1). On the other hand, the disturbed immunosurveillance allows neoplastic lymphoproliferations and solid cancers to develop, often promoted by viruses as EBV and CMV (Figures 2–**4**).

## Case reports

### Lymphomas

Patient A.III.1 is a 30-year-old female, who presented with antibody deficiency at age 15 and the diagnosis of CTLA-4 insufficiency was made at the age of 27 years. Additional complicating features included CNS involvement, psoriatic skin irritation, arthralgia, and a recurrent enteropathy, treated by steroids and immunoglobulin replacement therapy (IGRT).

By the age of 30 years she had lost 6 kg of weight during 3 months and multiple enlarged lymph nodes on both sides of the diaphragm were detected. Supraclavicular lymph node resection revealed a grade IV EBV-associated Hodgkin lymphoma with mixed cellularity (Figure 2). Laboratory findings showed a viral EBV load of 59,000 IU/ml blood.

The EBV viremia was treated with four courses of rituximab. The patient recently received her first course of AVD-Brentuximab and is currently stable.

Patient H.II.1 was a 21-year-old male, who presented with protracted diarrhea, ITP, and AIHA at the age of 10 years. The cytopenias became steroid-dependent and prompted intensive immunosuppression and finally splenectomy at the age of 20 years.

Moreover, he developed lymphocytic CNS lesions and recurrent generalized lymphadenopathies during his adolescence. Repeated biopsies revealed polyclonal cellularity in the lymph nodes and aplasia, fibrosis, and nodular lymphocytic aggregates in the bone marrow, compatible with an autoimmune lymphoproliferative syndrome-phenotype (Figure 1). Additional, he suffered from a cholestatic giant cell hepatitis at the age of 17 years (Figure 1).

At the age of 21 years, his EBV load had risen from 2,000 copies/ml to 8,400 copies/ml within 4 weeks, accompanied with high fevers and worsening clinical condition. He was admitted to intensive care due to progressive respiratory insufficiency, severe pancytopenia, and severe colitis. Despite immunosuppressive treatment with everolimus and prednisolone, rituximab, and high dose dexamethasone, the patient deteriorated and developed sepsis. Sequential therapy approaches with MMF, ATG, and G-CSF were made and quadruple-therapy for a concomitant atypical tuberculosis was initiated. Nonetheless, his condition worsened and he died 4 months after the onset of his EBV viremia. Pathology revealed post-mortem the diagnosis of an EBV-associated classic Hodgkin lymphoma with bone marrow infiltration (Figures 1, 2).

Patient B.II.3 is a 54-year-old male, in whom CTLA-4 insufficiency was revealed by family screening at the age of 49 (2). Fever, night sweats, and fatigue occurred just a few months later, EBV viral load began to rise, and a generalized lymphadenopathy was detected. In addition, pancytopenia developed in the context of a hemophagocytic syndrome. Laboratory values showed an IL2-receptor load of 44.141 U/ml and an EBV load of 297.000 copies/ml blood. Although a therapy with high dose corticosteroids, rituximab, and etoposide was initiated, his condition aggravated and he developed *Aspergillus fumigatus* sepsis. Aged 51 bone marrow biopsy revealed a classical Hodgkin lymphoma. With an adjusted chemotherapy protocol AVD (bleomycin was excluded due to aspergillosis) the patient reached clinical remission, subsequent bone marrow transplantation was successfully realized and the patient is in complete remission for more than 3 years.

Patient L.II.2 is a 20-year-old male, who initially presented with inguinal and axillary lymphadenopathy and severe pancolitis, at the age of 16 years. The diagnosis of lymphocyte-rich Hodgkin lymphoma was established based on an inguinal lymph node biopsy.

Laboratory values remained negative for EBV, but immunohistochemical staining was positive for CD15, CD30, EBV-LMP, and EBV *in situ*-hybridization. Histological work-up showed architectural effacement by a diffuse and partially nodular infiltrate of lymphocytes and histiocytes; these cells were interspersed with Reed-Sternberg cells (Figure 2).

The Hodgkin lymphoma was treated with three courses of ABVD chemotherapy (Euronet PHL-C1 2007), the colitis with corticosteroids, sirolimus, and belatacept and the hypogammaglobulinemia with IGRT. PET-CT at the first re-evaluation after 3 months showed, that the lymphoma was now in remission. He underwent matched unrelated bone marrow transplantation with reduced intensity 7 months after diagnosis and is now alive and well 2 years post-BMT.

Patient JJ.II.2 is a 31-year-old male, who presented with antibody deficiency at the age of 10 years (CVID Euroclass B+smB-CD21low TR high). In the course of his illness, he developed recurrent respiratory infections, intermittent cytopenia, renal impairment necessitating dialysis, fluctuating EBV levels, enteropathy, and bilateral granulomatous lesions in the lungs. At the age of 28 years a heterozygous mutation in *CTLA4* was detected.

A few months later, clinical assessment indicated a weak patient with enlarged inguinal lymph nodes accompanied by intermittent fevers and the diagnosis of a Hodgkin lymphoma with mixed cellularity including bone marrow infiltration was made; EBV-PCR detected a low positive result of 90 copies/ml blood. Complete remission was reached by six cycles of AVD protocol; the patient is alive and well more than 500 days after his initial cancer diagnosis without any signs of recurrence.

Patient MM.II.1 is a 40-year-old male, who initially presented with haemolytic anemia at the age of 14 years and diagnostic workup led to the diagnosis of a CVID (EUROClass B+smB-21low TR norm). Several pneumonias and recurrent autoimmune phenomena with haemolysis and thrombocytopenia occurred and were temporarily controlled by corticosteroids and azathioprine.

After 13 years of clinical remission, MM.II.1 presented with lymphadenopathy and B symptoms at the age of 33 years. His condition deteriorated rapidly and a diagnosis of an EBV-associated classical Hodgkin lymphoma (grade IIIB) was made based on cervical lymph node resection (Figure 2). Laboratory values showed an EBV load of <500 copies/ml and a CMV load of <1,000 copies/ml blood. Complete remission was reached with four cycles of ABVD chemotherapy protocol.

During remission, recurrent gastrointestinal irritations and relapses of a past encephalomyelitis occurred intermittently. So far, there are no hints for recurrence of the lymphoma and the patient remains in remission for 7 years.

Patient K.II.1 was a 52-year-old female, who was the first out of four patients with Non-Hodgkin-Lymphoma in our cohort. She was diagnosed with CVID during her twenties and received IGRT for decades. At the ages of 33 and 39 years hyper-cellularity and lymphocytic infiltrations were found in her bone marrow, but malignant cell growth could be ruled out. However, 6 years later at the age of 45 years, generalized lymph node enlargement appeared accompanied with fevers, night sweats, and weight loss. Cervical lymph node biopsy revealed clonal lymphoproliferation with typical features of a DLBCL with EBV association.

The lymphoma was treated with four courses of rituximab with only partial response, thus she received four cycles of R-CHOP 21 and could reach a complete remission.

After 4 years of remission, she developed recurrent abdominal and retroperitoneal lymphadenopathy, but biopsies negated malignant transformation. At the age of 51 years, her clinical condition deteriorated soon and finally the diagnosis of a non-EBV-associated DLBCL (grade IVb) was made based on an additional diffuse hepatic lesion biopsy. Pathology examination described compact atypical B cell infiltrate with a component of high reactive T cells. The non-EBV-associated DLBCL only responded partially to treatment with two cycles of R-CHOP, two cycles of R-DHAP, as well as one cycle of R-BEAM. In the end, she suffered from CMV-viremia and deceased due to pneumonia and gastric bleeding 7 months following the relapse at the age of 52 years.

Patient UU.III.3 was a 51-year-old female, who had severe and recurrent gastroenteritis for many years. Clinical records are rare and even genotyping could not be made prior her death, but family screening revealed a heterozygous *CTLA4* mutation in five out of seven siblings and in four out of her five children. At the age of 50 years she presented with inguinal lymphadenopathy accompanied with B symptoms and she was diagnosed with DLBCL based on inguinal lymph node resection.

Despite five cycles of R-CHOP, local radiotherapy, and radio-immune-therapy with ibritumomab-tiuxetan, the patient died 13 months after cancer diagnosis.

Patient CO.I.1 was a 62-year-old male, whose CVID diagnosis was first made at the age of 38 years and a heterozygous mutation in *CTLA4* was identified at the age of 61 years following clinical assessment. His clinical history followed a long history of recurrent but steroid-sensitive granulomatous infiltration in kidney, skin, lung, and conjunctivae. Finally, he complained of weight loss and fatigue over months at the age of 62. Biopsy of a hepatic lesion revealed morphological features of a DLBCL germline subtype. Immunohistochemical staining showed atypical lymphoid infiltrates, which were positive for CD20, Bcl6 and Bcl2, and negative staining for CD3, CD5, CD10, MUM-1, TdT, and EBERish.

The lymphoma was treated with three cycles of R-CHOP chemotherapy (two with reduced, one with full intensity), his health deteriorated and he deceased after a short and fulminant sepsis just 3 months after cancer onset.

Patient FF.II.1 was a 23-year-old male, who initially attracted clinical assessment at the age of 6 years and at the age of 16 years with treatment-dependent immune thrombocytopenia (ITP). He presented at age 22 with diffuse lymphadenopathy and in the years prior to his diagnosis he had benign lymphadenopathy with negative biopsies on multiple locations. At the time of his diagnosis, the lesions had increased in size, number, and PET-CT avidity prompting repeated biopsies. Those revealed typical features of a Burkitt lymphoma without EBV association. Laboratory values showed overall lymphopenic levels and negative EBV, CMV, and toxoplasma ranges. Immunohistochemical staining was positive for CD10, CD20, PAX5, c-MYC, and 100% for proliferation index Ki-67.

The lesions were refractory to four cycles of R-Hyper-CVAD and showed only a minimal response to two cycles of R-ICE. Next, he started treatment with rituximab and selinixor on study KPT330, but he was taken off the study because of disease progression with worsening thoracic and retroperitoneal lymphadenopathy. Ultimately, another therapy attempt was started following the DA-R-EPOCH protocol, but nonetheless the patient died of his progressive disease with thoracic and retroperitoneal lymphadenopathy at the age of 23 years.

### Gastric cancers

Patient B.II.4 is a 47-year old female, who had manifested with antibody deficiency, recurrent hypokalaemic periods due to severe diarrhea, and chronic renal failure most likely due to lymphocytic renal infiltrations during her early forties. Her *CTLA4* mutation was found at the age of 43 years by screening of 71 unrelated patients with CVID and enteropathy (2).

Two years prior her CTLA-4 insufficiency diagnosis, at the age of 41 years, she developed an EBV-associated and poorly differentiated gastric adenocarcinoma (type M, Figure 3), that was treated by radical mucosal resection. Nonetheless the cancer relapsed 3 years later—again EBV-associated (Figure 3)—and the patient finally underwent total gastrectomy at the age of 44 years and reached a complete remission; 19 lymph nodes were negative for cancer. Her clinical condition improved steadily under immunosuppressive therapy and today her intestinal symptoms are well-controlled.

Patient XX.II.1 is a 44-year-old male, who had manifested with a number of hospitalisations due to gastrointestinal symptoms such as appendicitis and severe diarrhea, that prompted many years on budesonide and anti-TNF-α treatment.

Gastroscopy at the age of 42 years showed severe atrophic gastritis with extensive intestinal metaplasia, a well-known risk-factors of gastric cancer (12). The diagnosis of an *in situ* adenocarcinoma was based on two tubular adenomas and extensive endoscopic resection of the lesions was performed (Figure 3). Laboratory values showed EBV load of 7,510 UI/ml blood and no CMV level, but PCR of biopsies were positive for EBV, CMV, and in presence of *H. pylori*.

He is receiving sirolimus, ustekinumab, and will start an antibiotic treatment for *H. pylori*. First control by gastroscopy 6 months after diagnosis showed two quiescent tubular adenomas with low grade dysplasia—gastrectomy might finally be performed when aggravation occurs.

Both patients who developed EBV-associated gastric cancer had received abatacept, a CTLA-4-Ig-fusion-protein, which is expected to improve especially the gastrointestinal symptoms in adult autoimmune enteropathy but could raise EBV serum levels (13). In fact, abatacept treatment has to be interrupted in patient B.II.4 after 7 months due to increased serum levels of EBV copies and rituximab treatment was needed, but nonetheless a relapse of gastric cancer developed 2 months after interruption and patient finally underwent total gastrectomy. However, patient B.II.4 resumed abatacept treatment 18 months after her relapse following mucosal healing for her severe CTLA4-associated enteropathy, reporting a repeated sustained clinical recovery of her bowel symptoms under abatacept until today. The second case (XX.II.1) stopped his therapy with abatacept after 3 months when EBV level rose and switched to sirolimus plus ustekinumab to control sustained intestinal symptoms with protracted diarrhea. EBV load decreased within few weeks after interruption of abatacept to <500 copies/ml blood.

Nonetheless both patients will need yearly follow-up by gastroscopy and carefully monitoring of EBV load.

Patient M.II.3 was a 35-year-old male, who suffered from chronic diarrhea since he was 10 years (5). Additional features were bacterial pneumonia and acute hepatitis with uncertain etiology around the age of 24 years, that needed corticosteroid pulse treatment. Around this time, he was diagnosed with CVID and started IGRT.

Acute gastritis mucosal lesions prompted recurrent endoscopies around the age of 28 years, and finally multiple biopsies at the age of 34 years revealed a poorly differentiated adenocarcinoma, a well-differentiated tubular adenocarcinoma and epithelium cells with CMV-associated antigen; no metastasis was found (Figure 3).

For treatment, he underwent total gastrectomy without perioperative chemotherapy, but protracted diarrhea and enterocolitis continued postoperatively for months despite immunosuppressive therapy. Recurrence of cancerous lesions has been monitored by gastroscopies, but he never fully recovered and died 25 months after gastrectomy following a *Klebsiella pneumonia*-induced sepsis.

Patient G.III.2 is a 25-year-old male, who had repeated gastrointestinal examinations and recurrent active gastritis over several years, which was initially diagnosed as Crohn's disease at the age of seven years (4). Additionally, he developed trilineage cytopenia and CT scan examination revealed interstitial pulmonary nodules. Despite aggressive immunosuppressive treatment he required partial colectomy at the age of 14 years, which improved his condition together with budesonide and anti-TNF-α treatment. However, his atrophic gastritis progressed and at the age of 17 years endoscopic biopsies revealed an early invasive adenocarcinoma without EBV or CMV association. The patient's treatment consisted of a total gastrectomy. Histopathology of the stomach revealed diffuse marked chronic active gastritis with multifocal areas of low to high grade dysplasia and an early invasive adenocarcinoma; 20 lymph nodes were negative for cancer. Remission was reached, but enterocolitis is still active and he requires intensive immunosuppressive therapy due to the neurological, hematological and respiratory involvement. Nonetheless, the patient is in complete remission for 7 years.

Patient CM.II.2 is a 29-year-old female, who has developed recurrent infections since the age of one year and was diagnosed with CVID at the age of 14 years, treated with IGRT.

At the age of 20 years, assessment of curious paraesthesia and muscle power decrease revealed a vitamin B12 deficiency-associated pernicious anemia. Following this diagnosis, gastroscopy discovered an early gastric adenoma (gastric type) with high grade dysplasia and without EBV or CMV association. Endoscopic submucosal dissection was made and she has been on regular follow-ups, which repetitively confirmed chronic active gastritis. Finally, at the age of 25 years, follow-up gastroscopy revealed a poorly differentiated gastric adenocarcinoma, again without viral association (Figure 3).

The treatment consisted of laparoscopic subtotal gastrectomy. Chemotherapy was not realized and the patient does not have any cancer relapse since more than 3 years and is still treated only by IGRT.

### Other malignancies

Patient CM.I.2 is a 75-year-old male, who has been asymptomatic during his life regarding PID-associated symptoms. At the age of 60 years, he presented with an axillary sarcoma and received operative excision without chemotherapy and reached clinical remission. Aged 75, he had developed proteinuria and urine protein electrophoresis revealed a monoclonal peak in beta globulin region. Finally, bone marrow biopsy verified the diagnosis of multiple myeloma.

Since his sister and niece (CM.II.2) have been diagnosed with CTLA-4 insufficiency a few months prior the patient's cancer diagnosis, genetic testing revealed the same heterozygous variant in *CTLA4*. Additional antibody test showed decreased serum levels.

The patient recently received chemotherapy with bortezomib and melphalan (in reduction due to renal insufficiency). Shortly after the chemotherapy he developed complications including pericardial and pleural effusions, he was admitted to intensive care, but he recovered well.

To continue treatment for multiple myeloma, he was transferred to a central hospital, where a kidney biopsy was made, which was immunohistochemically positive for amyloid p (Figure 4).

Patient CZ.II.2 is a 25-year-old female, who was initially diagnosed with relapsing-remitting Multiple Sclerosis (MS) at the age of 19 years and subsequently was noted to be hypogammaglobulinemic at 23 years. Her neurological symptoms were treated with several MS-targeting monoclonal antibodies and immunosuppressive therapies over the years, which were blamed for most of her other autoimmune features including interstitial lung disease, lymphocytic enterocolitis, and cytopenia, until finally a diagnosis of CTLA-4 insufficiency was made at the age of 24 years. She showed additionally impaired control of oncogenic viruses presenting with precancerous CIN II and VIN II.

Her melanoma (left-ear) was first noted on routine skin checks at the age of 24 years and was excised with clear margins (Figure 4). Seven months later, she noted an enlarged left post-auricular lymph node. A fine needle aspirate revealed a non-small cell malignancy suggestive of metastatic melanoma, which was confirmed with an excisional lymph node biopsy. PET-CT scan revealed avid lesions scattered in her lungs and brain. However, review of her old imaging suggested that these were more consistent with her previously noted inflammatory lesions.

She underwent a total left neck lymph node dissection, which revealed five involved lymph nodes out of a total of 22, with soft tissue deposits and intramuscular extension, confirming the diagnosis of an aggressive and unresectable stage III metastatic melanoma. Genetic testing revealed an *NRAS* mutation not amenable to BRAF immunotherapy. A follow-up PET-CT scan revealed new lesions involving her right psoas muscle and right axilla, with reductions in the previously noted lesions, on a slightly higher dose of steroids (Figure 4). Biopsies of both new lesions revealed granulomatous change, without evidence of melanoma. Recently, the patient has commenced abatacept therapy and remains on methotrexate and prednisolone. She is receiving radiation therapy to her neck and surrounding area.

## Discussion

Primary immunodeficiencies (PIDs) are known to confer an increased risk of cancer predisposition (14). This seems to be explained by the impaired immune surveillance of lymphocytes by the defective immune system. For patients with CVID an increased susceptibility to malignancies, such as gastric cancer and particularly lymphomas is known (15, 16). Interestingly, this observation is paralleled by our results in CTLA-4-insufficient patients, showing predominantly lymphoma and gastric cancer. Lymphomas are known to occur in patients with primary immunodeficiencies, possibly as a result of the lymphopenia present in most patients. Gastric cancers often arise from a chronic inflammatory tissue, which is a commonly known risk factor (12). Despite to that, malignancies like the multiple myeloma in older individuals should also be seen in consideration of immune senescence.

**Figure 5:**
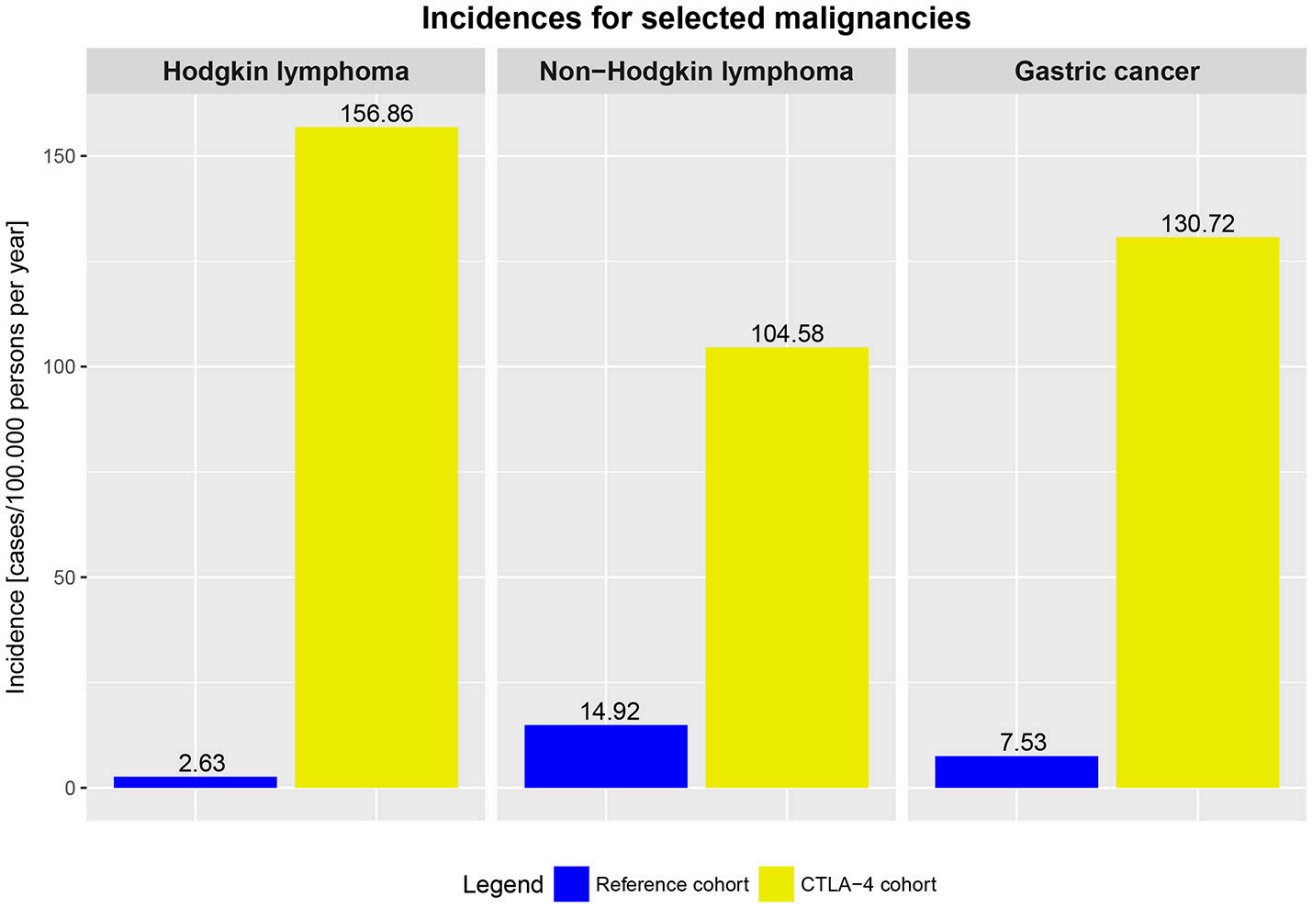
Incidences of selected malignancies in the averaged German and US population (reference cohort, general population, 2009–2014) and incidences in affected *CTLA4* mutation carriers (2009–2017).

Our findings demonstrate an elevated risk for malignancies of 12.9% in affected *CTLA4* mutation carriers. In addition, comparing the risk of cancer between the general population and affected *CTLA4* mutations carriers, affected *CTLA4* mutation carriers had a higher cancer rate per year (Figure 5).

**Figure 6:**
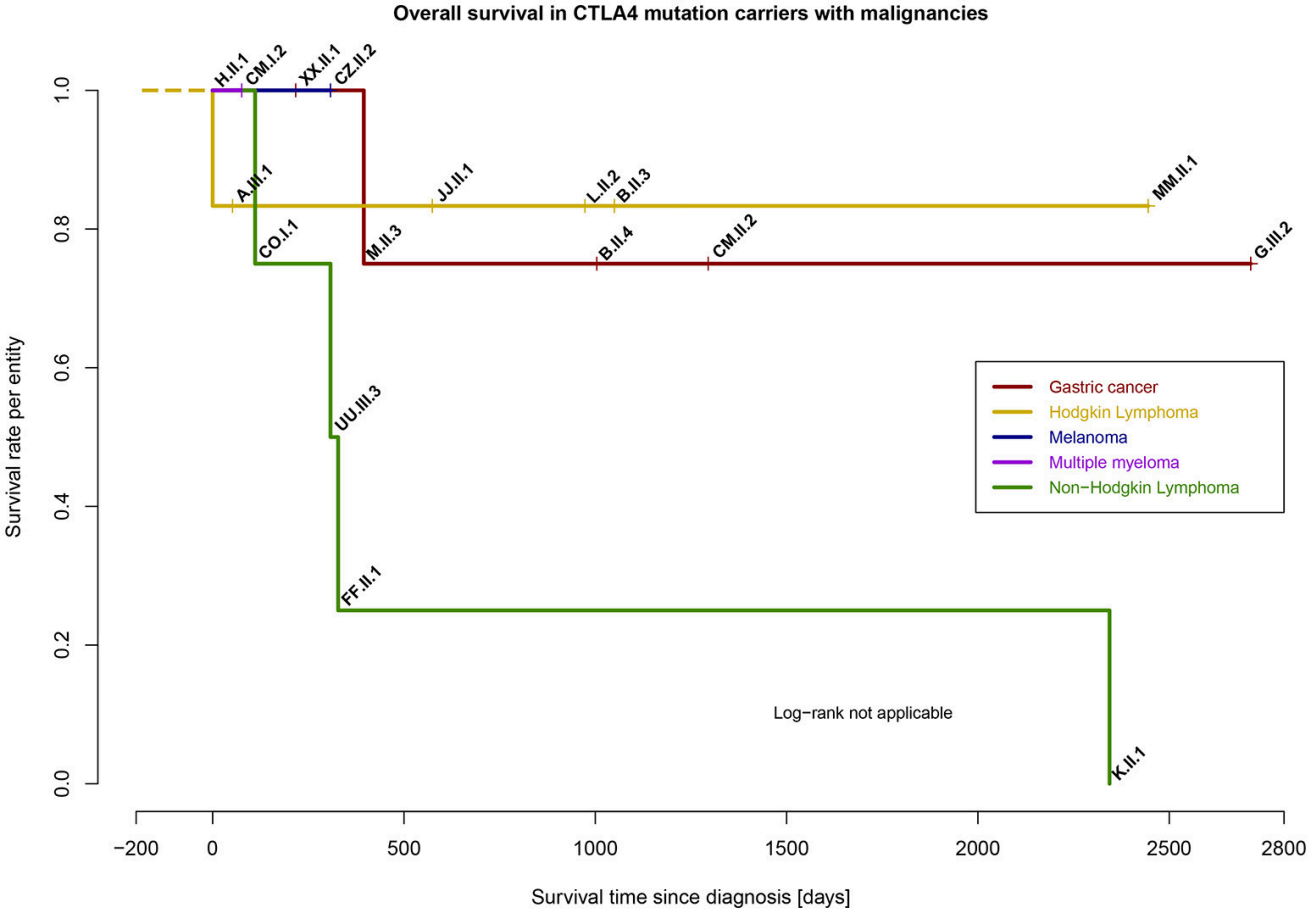
Survival rate per entity of malignancies in *CTLA4* mutation carriers (*n* = 17), time range from day of diagnosis to last follow-up (days).

The median time of survival since diagnosis of cancer was 361 days, however, this appears to vary dependent on entity (Figure 6). In 14 out of 17 patients, the diagnosis of CTLA-4 insufficiency was made prior the diagnosis of cancer. Special adjustment of cancer therapy because of the underlying PID was not reported, except in patient CZ.II.2, where additional immune dysregulatory features necessitated therapy with abatacept.

Lipopolysaccharide-responsive and beige-like anchor protein (LRBA) deficiency seems to be a phenocopy of CTLA-4 insufficiency (17, 18). In LRBA-deficient patients, CTLA-4 is transcribed normally, but impaired membrane trafficking induces an enhanced degradation of CTLA-4 in lysosomes (19). However, only a single report of gastric cancer was recently described for LRBA deficiency (20). The lack of reported malignancies in LRBA deficiency may result of the fact that the phenotype of LRBA deficiency is usually even more severe than that of CTLA-4 insufficiency and patients either die at an early age or receive HSCT, possibly prior to the development of any malignancy.

The reported melanoma in a CTLA-4-insufficient patient (CZ.II.2) is alerting and beyond our present understanding of the functional interaction between immune checkpoints and melanoma development. While the anti-CTLA-4 drug Ipilimumab acts to upregulate antitumor immunity and shows significant improvement in survival in metastatic melanoma, heterozygous *CTLA4* mutations should act similarly (21). Therapy is still pending but PD-1 inhibitors are being considered.

PD-1 blockade plus CTLA-4 insufficiency in patient CZ.II.2 should act like combined immune checkpoint inhibition, that had shown significant benefit on survival and CTLA4-Fc could attempt to decrease immune dysregulation (22). The possible deterioration of metastatic lesions under CTLA4-Fc should be considered, therefore the option of mono-chemotherapy with alloHSCT may be the best option if the patient's condition permits it.

Nine out of 17 patients had steroids (5–20 mg/d), one had additional anti-TNF-α treatment due to inflammatory bowel disease (XX.II.1), one had additional daclizumab due to MS (CZ.II.2), and two had received abatacept (CTLA4-Fc) additionally in order to ameliorate the CTLA-4 insufficiency. The occurrence of cancer in eight of 17 CTLA-4 patients without any immunosuppression indicates that there may be an intrinsic defect to control especially the herpes viruses, EBV, and CMV in CTLA-4 insufficient individuals. However, further studies and observations need to address the question whether or not immunosuppressive therapy may have been a secondary trigger for cancer development.

The increased cancer risk, combined with the observation of viral association, leads us to assume that a defective immune surveillance of chronically virus-infected cells and the reduced elimination of oncogenic viruses leads to deregulated cell growth. Decreased CTLA-4 expression results in uncontrolled proliferation of T cells with a possible overgrowth of autoreactive clones over e.g., EBV-specific T cell clones, as known for persons with HIV infection, but experimental data are missing (23).

As a result, monitoring of EBV and CMV replication in blood in patients with CTLA-4 insufficiency by PCR should be performed.

In general, EBV-infected memory B cells are controlled mainly by Naturel Killer (NK) and CD8+ T cells. The quantity and quality of the CD8+ T cell response to EBV are essential to control the infection (24). In some PID patients an underlying genetic disorder affecting T- and NK cell function results in failure to control EBV infection, predisposing for EBV-associated HLH and EBV-associated cancer (25). Recently, alerting high frequencies of asymptomatic EBV viremia in affected and unaffected individuals with *CTLA4* mutations were described, showing the clinical importance of further research about CTLA-4 signaling and EBV infection (26).

Since the functional outcome of NK and CD8+ T cells depends on a balance between activating and inhibitory signals, costimulatory molecules such as CTLA-4 should be considered to play a critical role in control of EBV infection. NK cells express CTLA-4, resulting in decreased IFN-y secretion upon engagement by the recombinant ligand CD80 (27). Notably, *CTLA4* mutation carrier NK cells show defective effector functions, but normal peripheral maturation (28). Furthermore, EBV-specific CD8+ T cells in EBV-associated DLBCL (*CTLA4* wildtype) are functionally impaired, hence, CTLA-4 insufficiency might push the oncogenic capacity of EBV (29).

Together, changes in the function and clonal diversity of NK and T cells due to CTLA-4 insufficiency might be further augmented by viral persistence and may lead to life-threatening EBV. Whether the increased risk, however, also extends to HPV-induced malignancies [as e.g. seen in ICOS-deficiency (30)] needs to be monitored in affected individuals, even more since similar precancerous lesions had been seen in patient CZ.II.2.

Whether abatacept treatment will decrease the risk to develop cancer in CTLA-4 insufficiency remains to be proven, as the substitution of CTLA4-Fc ameliorates the dysregulated immune response. Until then, HSCT should be the preferred option and should be considered early-on in the treatment strategy of malignancies in the context of CTLA-4 insufficiency.

Cancer predisposition in CTLA-4 insufficiency appears as a result of immune activation with chronic inflammation, failure to control oncogenic viruses or neoplastic cells by immunological means, plus an intrinsic T cell impairment due to the underlying genetic defect. These findings go along with the observation that individuals with PIDs have intrinsic abnormalities of the lymphoid system which result in an increased frequency of malignant cell growth (31). Moreover, recently published data supports this hypothesis of cancer predisposition: Hauck et al. discussed similarly and separated in intrinsic lymphoid abnormalities and extrinsic insufficient cancer immunosurveillance with failure to eliminate oncogenic viruses and chronic tissue inflammation (32). However, the spectrum of malignancies in patients with PIDs as well as in CTLA-4 insufficiency appears to be restricted and derived from the same molecular defect as the immunodeficiency itself; either in the same cell type that has been primarily affected by the immunodeficiency or in another cell type in which malignant transformation could occur secondarily and indirectly facilitated by the underlying PID (32).

## Author contributions

DE and CS, data collection and writing of the manuscript; AG, data collection; PA, LG-R, Y-JK, MM, ON, SOj, SOk A-CP, SSe, SSn, KW, and DW, contribution of patient data; PD, MS, EC, K-MK, J-MK, CM contribution of histologic images; BG, patient care, conception of the study, and writing of the manuscript.

### Conflict of interest statement

The authors declare that the research was conducted in the absence of any commercial or financial relationships that could be construed as a potential conflict of interest.

## Abbreviations

ABVD: Adriamycin (Doxorubicin), Bleomycin, Vinblastine, Dacarbazine
allo-HSCT: Allogeneic hematopoietic stem cell transplantation
BMT: Bone marrow transplantation
CIN: Cervical intraepithelial neoplasia
CVID: Common variable immunodeficiency
DA-R-EPOCH: Rituximab, Etoposide, Prednisolone, Oncovin, Cyclophosphamide, Hydroxychloroquine
HSCT: Hematopoietic stem cell transplantation
IGRT: Immunoglobulin replacement therapy
ITP: Immune thrombocytopenia
MS: Multiple Sclerosis
LRBA: Lipopolysaccharide-responsive and beige-like anchor protein
PBSC: Peripheral blood stem cells
PCR: Polymerase chain reaction
PET-CT: Positron-emission tomography and computer tomography
PID: Primary immunodeficiency
PD-1: Programmed cell death protein
R-BEAM: Rituximab, Camustin, Ara C, Etoposid, Melphalan
R-CHOP: Rituximab, Cyclophosphamide, Hydroxychloroquine (Doxorubicin), Vincristine (Oncovin), Prednisolone
R-CVAD: Rituximab, Cyclophosphamide, Vincristine, Adriamycin, Dexamethasone
R-DHAP: Rituximab, Dexamethasone, high Ara-C (Cytarabine), Cisplatin
R-ICE: Rituximab, Ifosfamide, carboplatin, etoposide
VIN: Vulvar intraepithelial neoplasia.
